# Unveiling the gastric microbiota: implications for gastric carcinogenesis, immune responses, and clinical prospects

**DOI:** 10.1186/s13046-024-03034-7

**Published:** 2024-04-19

**Authors:** Zhiyi Liu, Dachuan Zhang, Siyu Chen

**Affiliations:** 1 Department of Oncology, Xin Hua Hospital, School of Medicine, Shanghai Jiao Tong University, Shanghai, 200092 China; 2 Department of Pathophysiology, Key Laboratory of Cell Differentiation and Apoptosis of the Chinese Ministry of Education, Shanghai Jiao Tong University School of Medicine, Shanghai, 200025 China

**Keywords:** Gastric microbiota, Gastric carcinogenesis, Anti-tumor immunity, Immunotherapy

## Abstract

High-throughput sequencing has ushered in a paradigm shift in gastric microbiota, breaking the stereotype that the stomach is hostile to microorganisms beyond *H. pylori*. Recent attention directed toward the composition and functionality of this 'community' has shed light on its potential relevance in cancer. The microbial composition in the stomach of health displays host specificity which changes throughout a person's lifespan and is subject to both external and internal factors. Distinctive alterations in gastric microbiome signature are discernible at different stages of gastric precancerous lesions and malignancy. The robust microbes that dominate in gastric malignant tissue are intricately implicated in gastric cancer susceptibility, carcinogenesis, and the modulation of immunosurveillance and immune escape. These revelations offer fresh avenues for utilizing gastric microbiota as predictive biomarkers in clinical settings. Furthermore, inter-individual microbiota variations partially account for differential responses to cancer immunotherapy. In this review, we summarize current literature on the influence of the gastric microbiota on gastric carcinogenesis, anti-tumor immunity and immunotherapy, providing insights into potential clinical applications.

## Introduction

Gastric cancer (GC) is a very aggressive cancer and the third main cause of cancer-related mortality worldwide with more than 1 million diagnosed cases and 770,000 deaths globally in 2020 [1]. Approximately 40% of GC cases are identified at an advanced stage annually, with the 5-year survival rates for patients diagnosed in 2000 and 2014 merely 2.7% and 4.7%, respectively [2]. The infectious agent, *Helicobacter pylori* (*H. pylori*), is widely recognized as the most influential contributor to the onset of GC. An estimated 50% of people worldwide are infected with *H. pylori* [3] and approximately 90% of GC on a global scale result from the inflammation and damage caused by *H. pylori *infection [4]. Despite the well-established involvement of *H. pylori* in the process of gastric carcinogenesis, only fewer than 3% of *H. pylori*-infected patients eventually progress to GC, indicating the initial role of *H. pylori *rather than its exclusive influence on GC development. The scope of other contributing factors extends to genetic polymorphisms, environmental exposures, age, and gender [1, 5–7]. Recent progress has been made in the field, with the emergence of advanced next-generation sequencing technologies that unveiled the variations in diversity and community structure of gastric microbiota between individuals with good health and those afflicted with GC [8, 9]. These distinctions extend beyond the well-known *H. pylori* and include a broader range of microbial constituents, enlightening the potential role of these non-*H. pylori* gastric microbiota in the promotion of malignancy.

The microbiota encompasses a diverse array of bacteria, viruses, bacteriophages, fungi, and protozoa collectively shaping a complex ecosystem. These microbial communities, residing in various body habitats, including the oral cavity, gut, lung, skin and genitourinary tract, have been the subject of an extensive investigation regarding their contributions to maintaining homeostasis and their involvement in disease processes [10–14]. In particular, the mounting body of evidence highlights the role of the gut microbiota in driving cancer initiation and progression, orchestrating the intricate landscape of the tumor microenvironment (TME), and providing novel avenues for manipulating the microbiota to modulate anti-tumor immunity [15–19].

Owing to the relatively lower biomass of gastric microbiota, which quantifies merely 10^1^ to 10^3^ bacteria per gram of gastric content—significantly fewer than the 10^4^ to 10^7^ bacteria per gram found in the jejunum and ileum, and the 10^11^ to 10^12^ bacteria per gram in the colon, the gastric microbiota has received relatively limited attention in prior research [20]. Until recently, studies have conducted in vivo manipulations of gastric microbiota based on sequencing results, thus providing robust evidence to elucidate the causal relationship between perturbations in the gastric microbial ecosystem (referred to as "gastric dysbiosis") and the onset and progression of GC [21–23]. In this comprehensive review, we explore the emerging findings regarding the dynamic influence of gastric microbiota on GC and also address *H. pylori* as the most thoroughly investigated constituent of the microbiota, but are more inclined to discuss the evidence concerning its influence on the coexistence of other bacterial species in the stomach. We emphasize the pivotal role of gastric microbiota in the processes of gastric carcinogenesis, modulation of anti-tumor immune responses, and its potential implications in clinical application.

## Microbial composition in the stomach

Due to low biomass of the microbiota and the unique and challenging growth environment in gastric mucosa, methods for isolation and cultivation of the gastric flora, as well as those for strain identification via morphological, biochemical and serotype characteristics, are greatly constrained. Therefore, in early assessment of gastric microbial composition, cultivation-independent methods such as molecular fingerprinting have been widely used [24]. Among these, temperature gradient gel electrophoresis (TGGE) was the most popular technique, which separate microbial DNA into a series of bands for inter-species comparisons based on differential physical–chemical properties of DNA fragments. Apparently, TGGE's resolution to discriminate between microbial taxa is very limited, leading to marked underestimation of gastric microbiome richness [25]. Additionally, other molecular methods such as fluorescence in situ hybridization (FISH), microarrays, and quantitative PCR exhibit high sensitivity in detecting low-abundance species [26–28]. However, the requirement for species-specific oligonucleotide probes or primers prevented large-scale and unbiased analysis of the entire microbial community.

In the past decade, the development of next-generation sequencing (NGS) technologies has dramatically improved the precision and coverage of taxa identification, eventually enabling deep profiling of gastric microbiome, and dissecting their interactions with the host. Among the NGS methods, 16S rRNA sequencing has been widely used, because the 16S gene contains both the conserved regions supporting phylogenetic classification at the phylum level, and the rapidly evolving regions suitable for finer taxonomic resolution. However, 16S rRNA gene sequencing can only identify known species, with a resolution limited to the genus level, and losses all the functional information for each species. By contrast, metagenomics relies on whole genome shotgun sequencing, thus enabling higher resolution for species identification and functional analysis. NGS analysis often synergize with other multi-omics methodologies such as metabolomics and proteomics, providing a comprehensive understanding of the structure and functions of gastric microbiota in health and disease [25, 29].

*H. pylori* initially identified in the 1980s was regarded as a milestone to investigate gastric microenvironment. Subsequent advancements in detection technologies have enabled the meticulous tracking of the microbial ecosystem and unveiled the microbial profile of the stomach (Table 1). This groundbreaking progress challenges the longstanding stereotype that the gastric mucosa is fundamentally sterile attributed to its inhospitable condition [24, 30].

**Table 1:** Gastric microbial composition in health

Age	Main stomach taxonomic structure in health/control sample	Gastric sample type	Subjects	Country	Method	Ref
Infant (1 hour after birth)	*Lactobacillus* (vaginally delivery), *Ureaplasma* (premature birth)	Gastric fluid	29	America	16S rRNA sequencing	[31]
27 + / − 0.5 weeks (gestational age) preterm neonates	Phyla: Firmicutes, Proteobacteria, Tenericute, Actinobacteria in week 1 Firmicutes, Proteobacteria in week 4 Genera: *Staphylococcus*, *Streptococcus, Ureaplasma, Neisseria, Haemophilus, Klesbsiella*	Gastric aspirates	12	America	16S rRNA sequencing	[32]
Preterm neonates	Gram-positive: *Staphylococcus, Lactobacillus* and *Enterococcus*, Gram-negative: *Serratia, Klebsiella* and *Escherichia*	Gastric fluid	13	Spain	16S rRNA sequencing	[33]
27.7 + / − 2.8 weeks Preterm neonates	*Bacteroides* spp., *Lactobacillus* spp.*, Escherichia coli, Staphylococcus aureus, Streptococcus pneumoniae, Bifidobacterium* spp.(breast-fed neonate)	Gastric aspirates	22	America	16S rRNA-based Denaturing gradient gel electrophoresis	[34]
Adult	Proteobacteria, Firmicutes, Bacteroidetes, Actinobacteria, Fusobacteria	Gastric mucosa	HC:23	America	16S rRNA sequencing	[30]
Adult	Phyla: Proteobacteria, Bacteroidetes, Firmicutes, Actinobacteria, Fusobacteria Genera:* Streptococcus, Prevotella, Neisseria, Haemophilus, Porphyromonas*	Gastric mucosa	HC:5 non-NSAID gastritis:5	Hong Kong (China)	16S rRNA sequencing	[35]
Adult	Phyla: Proteobacteria, Bacteroidetes, Firmicutes, Fusobacteria, Actinobacteria Genera: *Neisseria*, *Prevotella, f;[paraprevotellaceae]-g;[Prevotella], Haemophilus, Fusobacterium, Streptococcus, Veillonella, Capnocytophage, Leptotrichia*	Gastric mucosa	HC:7	Japan	16S rRNA sequencing	[36]
Adult	Phyla: Firmicutes, Bacteroidetes, Proteobacteria, Actinobacteria, Fusobacteria Genera: *Streptococcus*, *Prevotella*, *Veillonella*, *Fusobacterium*, *Gemella*, *Neisseria*,* Haemophilus*	Gastric mucosa	HC:171 Non-atrophic *H. pylori* gastritis:33 AG:12 antral chemical gastritis:61	Sweden	16S rRNA sequencing	[37]
Adult	*Enterococcus, Pseudomonas, Streptococcus, Staphylococcus, Stomatococcus*	Gastric mucosa	HC:5 CG:8	Sweden	Temporal temperature gradient gel electrophoresis	[24]
Adult	Phyla:Firmicutes, Proteobacteria, Bacteroidetes, Fusobacteria, Actinobacteria Genera:* Streptococcus, Prevotella, Pseudomonas, Fusobacterium, Gemella, Neisseria, Veillonell*	Gastric mucosa	HC:21	German	16S rRNA sequencing	[38]

*Abbreviation*: *GC* Gastric cancer, *HC* Healthy control, *AG* Atrophic gastritis, *NSAID* Non-steroidal anti-Inflammatory drugs

### Initial establishment of gastric microbial community

**Fig.1:**
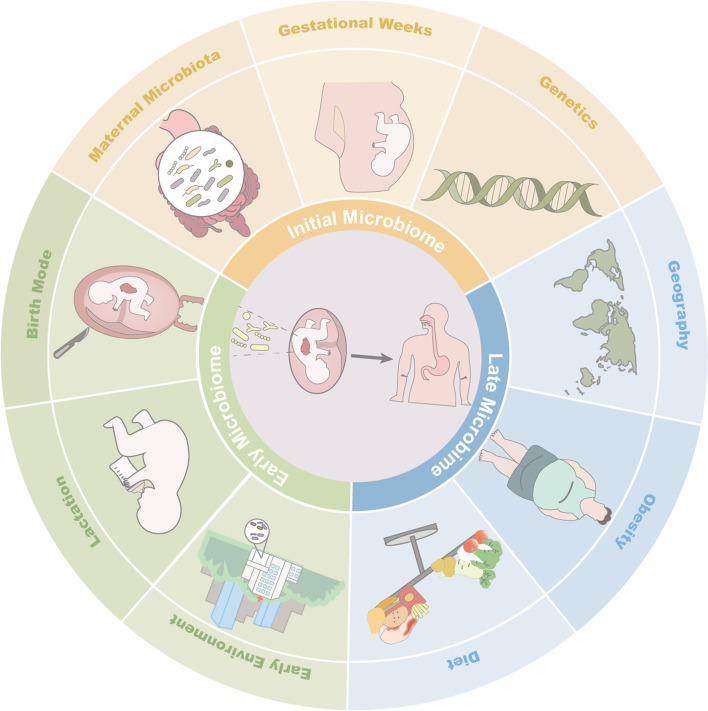
A multitude of factors influence the colonization of the gastric microbiota from prenatal development through adulthood. In the prenatal stage, the establishment of the initial gastric microbiome is shaped by factors such as the maternal microbiota, duration of gestation, and individual genetic makeup. Subsequently, during the neonatal period, variables including delivery mode, lactation method, and environmental exposures exert additional influence on early-life microbial composition. As individuals’s transition into adulthood, dietary preferences, obesity status, and geographic location persist as pivotal determinants in molding the architecture of the gastric microbiome

The stomach is not as bacteria-free as expected after birth. Microbes are present in the gastric aspirate from newborn infants, suggesting that the initial establishment of the gastric microbial community may occur at delivery or even earlier. The microbial composition of the neonatal stomach displays variability and potentially bears a connection to the mode of delivery (Fig. 1). Term neonates with vaginal delivery have significantly higher gastric microorganism abundance than those born by cesarean section. Additionally, the identification of the predominant constituent in the neonatal gastric microbial community highlights *Lactobacillus Crispatus*as the prevailing species within this ecosystem. This bacterium is crucial for maintaining vaginal health and becomes enriched within the vaginal mucosa during pregnancy [31, 39]. Whereas in preterm infants, the initial colonizers in the stomach are instead characterized by a high abundance of *Ureaplasma* [31, 32]. The detection of *Ureaplasm* from the maternal placenta and amniotic fluid predicts higher risk of preterm birth and neonatal mortality, and the persistent colonization of *Ureaplasm *in neonates may be associated with systemic infection and congenital diseases such as bronchopulmonary dysplasia [40]. However, *Ureaplasm *appeared only in the gastric aspirate of neonates in the first week, but disappeared afterward, probably due to intolerance of the gastric environment and prompt intervention with antibiotics [32]. Subsequent exposure to the environment and breast milk feeding flourish more bacteria species colonization in the stomach. A study involving 13 one-month-old preterm infants collected gastric contents and identified a predominant presence of *Staphylococcus*, *Lactobacillus*, and *Serratia*. These microbial components are recognized as significant constituents of breast milk [33, 41]. In addition, gastric microbial species of these preterm infants with long-term hospitalization were observed to overlap with a large proportion of nosocomial infection-associated bacteria from the hospital environment, such as *Staphylococcus aureus*, *Staphylococcus epidermidis*, *Enterococcus faecium*, *Neisseria*, *Klebsiella pneumoniae*, and *Escherichia coli* [32–34]. Environment factors seem to act as an essential selector for inhabitants of the stomach, and even for twins, the initial development of microbial community varies according to different exposure to environmental flora [42].

The initial gastric colonizers can shape the local and systemic immunity in early life. In animal models, it has been demonstrated that the offspring of mothers who were given antibiotics during pregnancy and breastfeeding had a decrease in microbial diversity in gastric contents accompanied by elevated IgG and IgM [43]. Consistently, Trevisi et al. also indicated that high-quality and complex initial microbial residence in the stomach not only favor oxyntic mucosa maturation and development of acid secretion but also mediate gastric immunity by modulating interferon response, antibodies production and immune cells maturation [44].

### Gastric microbial composition in Health

As early as 2000, Monstein et al. initially described gastric “indigenous” microbiota other than *H. pylori* by temporal temperature gradient gel electrophoresis(TGGE). *Enterococcus*, *Pseudomonas*, *Streptococcus, Staphylococcus*, and *Stomatococcus*were detected as major colonized members of genera in the stomach [24]. In 2005, a 16S rDNA sequencing of human stomach bacterial profile in America uncovered 128 phylotypes belonging to five predominant phyla which are Proteobacteria, Firmicutes, Bacteroidetes, Actinobacteria and Fusobacteria, accounting for over 90% of the total [30]. Generally, consistent phylum structures were identified in subsequent research in Hong Kong and Japan, whereas the American population showed a higher Firmicutes/Bacteroidetes ratio compared with Asians [35, 36]. High Firmicutes/Bacteroidetes ratio was previously mentioned in both animal study and clinical trial as a biomarker of gut dysbiosis in obesity [45, 46], and mice that were fed high-fiber or high-fat food exhibited overrepresented Bacteroidetes and Firmicutes for each [47, 48]. This inter-ethnic difference in Firmicutes/Bacteroidetes ratios within the gastric microbiota may be influenced by dietary habits. Moreover, a more extensive sequencing was performed in Sweden, an area with low *H. pylori *prevalence. This study recruited 171 healthy subjects and unveiled the taxonomic composition of their gastric microbial community. Sweden's population showed an unexpectedly low abundance of Proteobacteria [37], which is noteworthy as *H. pylori* falls within this bacterial clade. Genetic variants and external factors within the Swedish population may contribute to the development of a gastric environment that is less conducive to Proteobacteria colonization, thereby leading to a decreased incidence of *H. pylori* infections.

The most prevalent gastric mucosa-associated genera frequently described in the scientific literature are *Neisseria, Prevotell, Haemophilus*, *Fusobacterium*, *Streptococcus,* and *Veillonella*, though the abundance of each varies among subjects [24, 35–37]. *Neisseria*, *Haemophilus*, *Fusobacterium* and some species of *Streptococcus* and *Prevotella* are typical oral bacteria [8, 36, 49]. The swallowing action allows the oral microbiota to "seed" the lower GI tract. It is noteworthy that these prevailing genera within the stomach are scarcely detected in the lower gastrointestinal (GI) tract, which is primarily characterized by microbial communities dominated by *Ruminococcus*, *Faecalibacterium*, and *Bacteroides* [36, 38]. This large difference highlights the inaccuracy of regarding the lower GI and fecal microbiota as representative of the entire GI microbiota and underscores the importance of studying the GI microbiota by compartmentalization. Nonetheless, the outcomes of sequencing solely capture the configuration of the gastric microbiota at a specific juncture within the digestive process. It remains elusive to ascertain whether these microbial communities represent transient contamination or persistent colonization.

## Gastric microbiome, *H. pylori* and Gastric cancer

GC, a complex and multifactorial disease, is known to develop through a multistep progression involving a series of pathological processes referred to as Correa’s cascade. The sequence of events typically begins with superficial gastritis (SG), followed by chronic atrophic gastritis (CAG), intestinal metaplasia (IM), and dysplasia, ultimately culminating in the occurrence of GC [50]. These sequential pathological changes contribute to the stepwise transformation of normal gastric mucosa into malignant tissue. In addition to *H. pylori* infection as the main driving factor, characteristic changes in the gastric microbiota concomitant with this process were observed (Table 2).

**Table 2:** gastric microbial diversity and composition of gastric cancer patients

Subject /Sample Size	Gastric sample type	Country	Method	Gastric microbial diversity alternation in diseases	Gastric microbial composition alternation	Ref
12 GC VS 20 functional Dyspepsia	Gastric tissue	Malaysia	16S rRNA sequencing	GC: ↑	GC: *Lactococcus*, *Veilonella*, *Fusobacteriaceae* (*Fusobacterium*), *Leptotrichia* ↑	[8]
268 GC VS 288 control	Gastric mucosa	Korea	16S rRNA sequencing	GC: ↓	GC: *Helicobacter*, *Propionibacterium acnes*, *Prevotella copri* ↑ Control: *Lactococcus lactis* ↑	[9]
17 SG VS 10 AG VS 5 GIN VS 15 GC	Gastric mucosa	China	16S rRNA sequencing	NS	GC: *Slackia*, *Selenomona*s, *Bergeyella, Capnocytopha*, *Parvimonas, Eikenella*, *Prevotella-2*, *Kroppenstedtia*, *Lentibacillus*, *Oceanobacillus* ↑ GIN: *Romboutsia*, *Fusicatenibacte*r, *Prevotellaceae-Ga6A1-group, Intestinimonas* ↑	[22]
20 HC VS 20 Gastritis VS 40 AG VS 40 IM VS 48 GC	Gastric mucosa	Mongolia	16S rRNA sequencing	HC > IM > GC > gastritis > AG	*H. Pylori*(-) GC:*Lactobacilli* and *Enterococci *↑ *H. pylori*( ±) GC:*Carnobacterium*, Glutamicibacter, *Paeniglutamicibacter*, *Fusobacterium*, *Parvimonas *↑	[23]
17 SG VS 16 IM (low-risk) VS 6 IM (high-risk) VS 4 EGN	Gastric antral biopsies	Singapore	16S rRNA sequencing	NS	EGN: Proteobacteria(genus *Proteus*), *H. pylori *↑ Bacteroidetes(S24-7 group), *Bfidobacteria*, *Lactobacillu*s ↓ High-risk IM: *H. pylori*, *Actinomyces*, *Moryella *↑ Low-risk IM: *Phyllobacteriaceae*, *Enhydrobacter*, *Moryell*a ↑ *Bifidobacteria*, *Lactobacillus *↓	[51]
30 HC VS 21 CG VS 27 IM VS IN VS 29 GC	Gastric mucosa	China	16S rRNA sequencing	HC > CG > IM > GC	IM and GC: Gram-positive and anaerobic bacteria ↑ IN, IM and GC: *Nitrospira*e ↓	[52]
21 SG VS 23 AG VS 17 IM VS 20 GC from Xi an 56 SG VS 51 AG VS 19 GC from Hohhot	Gastric mucosa	China	16S rRNA sequencing	IM and GC: ↓	GC: *Peptostreptococcus stomatis*, *Streptococcus anginosus*, *Parvimonas micra*, *Slackia exigua*, *Dialister pneumosintes* ↑	[53]
61 SG VS 55 IM VS 64 GC	Gastric mucosa and Gastric fluid	China	16S rRNA sequencing	GC: ↓	GC: *Lactobacillus*, *Veillonella*, *Gemella* ↑	[54]
9 Gastritis VS 7 IM VS 11 GC	Gastric mucosa	China	16S rRNA sequencing	/	GC: *Clostridium colicanis*, *Fusobacterium nucleatum* and *Lactobacillus species* ↑ *H. pylroi*-negative patients.: *Burkholderia*, *Enterobacter*, and *Leclercia* ↑	[55]
89 IM VS 89 matched controls	Gastric mucosa	America	Shotgun metagenomic sequencing	NS	IM: *Johnsonella ignava*, *Actinomyces* sp. *oral taxon 448*, *Prevotella baroniae*, *Filifactor alocis*, *Veillonella* sp. *oral taxon 780*, *Leptotrichia goodfellowii* ↑	[56]
54 GC VS 81 CG	Gastric mucosa	Portuguese, China and Mexico	16S rRNA sequencing	GC: ↓	GC: phylum level: Firmicutes, Actinobacteria and non-*Helicobacter* Proteobacteria ↑; Genus level: *Citrobacter*, *Clostridium*, *Lactobacillus*, *Achromobacter*, *Rhodococcus*, *Phyllobacterium* ↑	[57]
230 Normal tissues VS 247 peritumoral tissues VS 229 tumoral tissues	Gastric tissue	China	16S rRNA sequencing	Normal > Tumor > Peritumoral tissue	Tumor: *Prevotella melaninogenica*, *Streptococcus anginosus*, *Propionibacterium acnes*↑ *H. pylori*, *Prevotella copri*, *Bacteroides uniformis* ↓	[58]
10 high GC risk VS 10 low GC risk	Gastric biopsy samples	Colombia	Whole metagenomic shotgun sequencing	NS	high GC risk: *Staphylococus*, *Streptococcus*, *Bacillus*, *Aspergillus*, and *Siphoviridae*↑	[59]
53 tumorous tissue VS 53 adjacent gastric tissue	Gastric mucosa	America	16S rRNA sequencing	Tumor: ↑	worse overall survival in GC: *Fusobacterium* and *Prevotella* ↑	[60]
62 cancerous tissues VS 62 neiboring non-cancerous tissues	Gastric mucosa	China	16S rRNA sequencing	Cancerous tissues: ↑	cancerous tissues: *Fusobacterium*, *Streptococcus*, *Peptostreptococcus*↑ non-cancerous tissues: *Lactococcus lactis*, *Lactobacillus brevis* ↑	[61]
25 *H. pylori*(-) SG VS 34 GC	Gastric mucosa	China	16S rRNA sequencing	NS	CG: *Lactobacillus, Streptococcus, Citrobacter, Klebsiella* ↑ *H. pylori*(-) GC: *Pseudomonadales, Betaproteobacteria*, *Gammaproteobacteria *↑ *H. pylori*( +) GC: *Campylobacterales* ↑	[62]
18 GC tumor samples VS 78 paracancerous samples VS 64 SG samples	Gastric mucosa	China	16S rRNA sequencing	GC: ↓	GC: *Dialister* spp.*, Helicobacter* spp.*, Lactobacillus* spp.*, Rhodococcus* spp.*, Rudaea* spp.*, Sediminibacterium* spp. ↑ tumor tissue: *Bradyrhizobium* spp., *Mesorhizobium* spp.↓	[63]
37 tumor tissues VS 37 adjacent non-tumor tissues	Gastric mucosa	China	16S rRNA sequencing	Tumor tissue: ↑	tumor tissues: *Lactobacillus, Streptococcus*, *Acinetobacter, Prevotella, Sphingomonas, Bacteroides, Fusobacterium, Comamonas, Empedobacter, Faecalibacterium* ↑ non-tumor tissues: *Helicobacter* ↑	[64]
53 GC VS 30 CG	Gastric mucosa	China	16S rRNA sequencing	GC: ↓	GC: *Oceanobacter*, *Methylobacterium*, and *Syntrophomona *↑	[65]
16 distal GC (normal and tumor tissue) VS 13 proximal GC (normal and tumor tissue)	Gastric tissue	China	16S rRNA sequencing	Tumor tissue: ↑	tumor: *Helicobacter*↓, *Lactobacillus* and *Muribaculaceae *↑ proximal GC: *Rikenellaceae_RC9_gut_group*, *Porphyromonas*, *Catonella*, *Proteus*, *Oribacterium*, and *Moraxella *↑ distal GC: *Methylobacterium_ Methylorubrum *↑	[66]

*Abbreviation*: *GC* Gastric cancer, *SG* Superficial gastritis, *AG* Atrophic gastritis, *GIN* Gastric intraepithelial neoplasia, *NS* Not Significant, *HC* Healthy control, *IM* Intestinal metaplasia

### Gastric microbiome in gastric carcinogenesis

#### Gastric microbial diversity in gastric carcinogenesis

Recent studies have investigated the variances in gastric microbial diversity across Correa's cascade, ranging from healthy controls (HC) to SG, AG, IM, and ultimately GC [21, 22, 38, 49–52]. Among these investigations, two studies did not observe significant differences in gastric microbial diversity among these subgroups [22, 51]. Other larger-scale studies reported significantly reduced microbial richness and diversity in GC when compared with SG or HC groups. Whereas the discrimination of gastric flora diversity between GC and precancerous lesions (IM and dysplasia) did not achieve statistical significance in these studies [53, 54]. Microbial diversity may undergo a substantial decrease between the AG and IM stages [67]. As shown in previous studies, *H. pylori *eradication significantly decreased the risk of GC whereas its impact is constrained in patients with IM and dysplasia [68, 69]. This indicated that the optimal therapeutic window for antibiotic intervention along Correa's cascade lies before the transition from AG to IM and this timepoint may be correlated with the observed changes in the microbial composition and ecosystem dynamics within the gastric environment.

#### Gastric microbial composition in gastric carcinogenesis

In parallel, the variations in the composition of the microbiota along the spectrum spanning from healthy gastric mucosa to GC were explored in several studies (Table 2). Through inter-comparison between the gastric microbiome of GC, pre-cancerous stages and normal tissue, these investigations unveiled the distinctive dysbiosis that characterizes gastric carcinogenic progress. A noteworthy observation is that gastric microbial composition is significantly influenced by hypochlorhydria, a main pathological manifestation of atrophy. This alteration in gastric acidity may result in increased vulnerability to colonization by microorganisms from other origins, primarily the oral cavity and intestine [33, 43, 48, 50, 53, 54].

For instance, a prospective cohort study undertaken by Sung et al. recruited 587 *H. pylori*-positive patients. They reported the presence of a specific assembly of oral bacteria, including *Prevotella*, *Rothia*, *Peptostreptococcus*, *Parvimonas* and *Streptococcus*, which were implicated in the occurrence and prolonged course of GA and IM [70]. A similar enrichment of oral microbes was found in a case–control study performed on 89 IM patients, where the abundance of *Johnsonella ignava*, *Peptostreptococcus stomatis*, *Neisseria elongata*, and *Neisseria flavescens *was found to be significantly higher in IM cases [56]. Moreover, an insightful study was conducted on a well-defined cohort, recruiting 34 cases of SC, 20 cases of AG, 5 cases of gastric intraepithelial neoplasia(GIN), and 15 cases of intestinal-type GC. Remarkably, they revealed a noticeable increase in the abundance of specific oral bacteria, including *Slackia*, *Selenomonas*, *Bergeyella*, and *Capnocytophaga*, which continuously increased from SG to GC. Concurrently, intestinal bacteria including *Romboutsia, Fusicatenibacter*, *Prevotellaceae-Ga6A1-group* and *Intestinimonas* were observed to be overabundant in GIN patients [22].

The predominance of these “non-indigenous colonizers” persists in GC which may significantly characterize malignant transformation. Coker et al. detected distinct Operational Taxonomic Units (OTUs) that were associated with five oral-derived species, including *Peptostreptococcus stomatis*, *Streptococcus anginosus*, *Parvimonas micra*, *Lackia exigua*, and *Dialister pneumosintes*, which can significantly distinguish GC from SG [53]. In another investigation where 54 GC patients and 81 chronic gastritis patients were enrolled, GC-representative genera exhibited a remarkable overabundance of intestinal commensals such as *Citrobacter*, *Clostridium*, *Lactobacillus*, *Achromobacter* and *Rhodococcus* [57]. Even by autologous comparison, tumor microhabitats were characterized by the discernible increase of oral pathogen, *Prevotella melaninogenica*, *Streptococcus anginosus* and skin pathogen *Propionibacterium acnes* (*P.acnes*), setting them apart from both paired paracancerous and normal tissues [58].

#### Gastric microbiota as predictive markers in gastric cancer

The recognition of microbial features during gastric carcinogenesis instigates exploration into the correlation between gastric microbiota and the multifaceted aspects of GC, such as risk, progression and prognosis. A Korean study, comprising a cohort of 268 GC patients and 288 controls, identified *H. pylori*, *P.acnes* and *Prevotella copri *as significant risk factors for GC, as indicated by odds ratios (ORs) of 1.86, 4.7 and 2.54 respectively [9]. Concurrently, another case–control study employed Whole metagenomic shotgun sequencing (WMS) on the gastric biopsy samples, which were obtained from a cohort of Colombian individuals, including both high-risk (*n* = 10) and low-risk (*n* = 10) populations for GC. The results revealed that the typical soil bacteria including *Bacillus*, *Actinomyces* and *Arthrobacter* spp., and *Keratinibaculum* spp. that was over representative in males, as well as skin commensal *Staphylococcus* and oral microbe *Streptococcus*, associated with an increased risk of GC [59].

In addition, a prospective longitudinal study was conducted to investigate the predictive value of gastric microbiota in GC progression. This comprehensive investigation involved 43 participants who underwent initial gastroscopy, followed by regular monitoring through 1–2 yearly gastroscopies over a minimum duration of 5 years. The results showed that patients who progressed from IM to early gastric neoplasia (EGN) during the study period had significantly higher relative abundance of *Moryella* genus and *Vibro *genus at baseline, compared to those who did not exhibit such progression [51].

Regarding the prognostic assessment of GC based on the gastric microbiome, multiple retrospective studies delved into distinctions in gastric microbial composition between GC patients with favorable and unfavorable prognoses. Several investigations highlighted the association between heightened levels of *Fusobacterium nucleatum* (*F. nucleatum*) in tumor samples and unfavorable prognostic outcomes among GC patients [60, 71, 72]. Notably, in Lauren’s diffuse-type GC, *F. nucleatum* positivity was found to be associated with markedly diminished overall survival rates. The abundance of *F. nucleatum* demonstrated a positive correlation with patient age, although it exhibited no significant associations with gender, *H. pylori *infection status, tumor stage, or tumor location [71]. Survival analysis from an additional cohort study has indicated that colonization of *F. nucleatum* species correlates with poorer prognosis among late-stage GC patients with *H. pylori *positivity [72]. These findings were further corroborated by an additional study showing that the presence of *F. nucleatum* and *Prevotella *species in tumor specimens has most significant impact on prognosis [60]. Furthermore, emerging evidence has also suggested potential correlations between specific bacterial species and favorable prognoses in GC. Using weighted gene co-expression network analysis (WGCNA), a recent study unveiled that GC patients exhibiting higher microbial diversity experienced poorer outcomes compared to those with lower microbial diversity. However, *Tissierella *enrichment was frequently observed in TP53 wild-type tumors and was associated with a favorable prognosis in various tumor types including GC, breast cancer and lung adenocarcinoma [73]. Another study revealed that GC patients with favorable prognoses displayed elevated *H. pylori* abundance alongside a decrease in *Halomonas* and *Shewanella *abundance [74]. Moreover, the presence of *Asinibacterium *in non-tumor adjacent tissues correlated with improved overall survival in GC patients, suggesting a potential influence of the gastric microenvironment on the pathophysiology of GC [60].

### Role of the gastric microbiota in gastric carcinogenesis

#### *H. pylori* in gastric carcinogenesis through the “hit and run” mechanism

**Fig.2:**
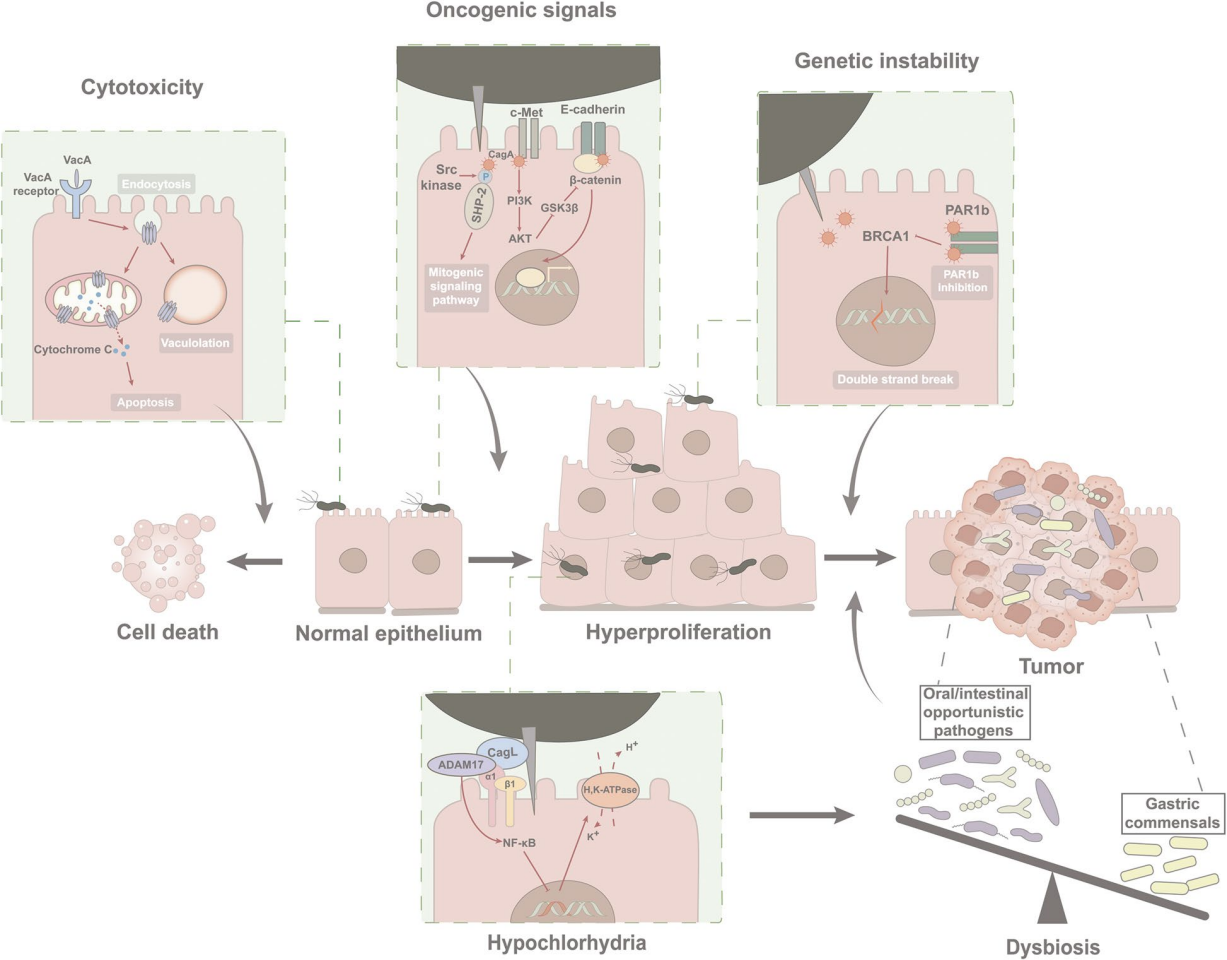
The "hit and run" mechanism posits a potential role for *H. pylori* in gastric carcinogenesis. The virulence factor VacA is internalized upon binding to cell surface receptors on host cells. This interaction initiates cell vacuolation and the subsequent release of cytochrome c from mitochondria within the cells, ultimately leading to cytotoxicity and cell death. The virulence factor CagA is translocated into the host cell through the T4SS. CagA drives cell hyperproliferation through three key mechanisms: It is phosphorylated by Src kinase, leading to the activation of mitogenic signaling pathways. CagA triggers the activation of c-Met and initiates the PI3K/Akt signaling cascade. CagA interacts with E-cadherin, disrupting the E-cadherin/β-catenin complex, thereby activating the Wnt signaling pathway. These pathways converge to induce the accumulation of β-catenin, which in turn may facilitate the overexpression of pro-oncogenic genes. The inhibition of PAR1b by CagA induces BRCAness, leading to double-strand breaks in the host cell. And CagL induces the separation of ADAM17 from α5β1 integrins, which subsequently mediates the inhibition of H, K-ATPase expression through NF-κB inhibitory binding, resulting in hyposecretion of gastric acid. This hypochlorhydria triggers gastric dysbiosis. The genetic instability and dysbiosis may replace *H. pylori* in maintaining subsequent tumor phenotype and promoting tumor progression

*H. pylori, *a known Class I carcinogen in the development of GC, exerts a significant predisposing effect on carcinogenesis by employing various virulence factors [75]. Its remarkable feature of urease synthesis enables it to thrive within the acidic environment of the stomach. The primary mechanism underlying *H. pylori*-induced carcinogenesis involves the expression of two oncogenic effector proteins: vacuolating cytotoxin A (VacA) and cytotoxin-associated gene A(CagA) protein [76] (Fig. 2).

VacA, a potent vacuolating cytotoxin present in all *H. pylori *strains, is capable of inducing vacuolation in host cells. VacA-forming pores can target mitochondria, causing depolarization of the mitochondrial transmembrane potential and subsequent mitochondrial dysfunction. This event triggers the release of cytochrome c from the mitochondria, which ultimately leads to the initiation of the apoptotic pathway [77]. The cag pathogenicity island (cagPAI) contains genes responsible for the expression of a type IV secretion system (T4SS) and the oncogenic protein CagA [78]. Upon the action of T4SS, *H. pylori *translocates CagA into the cytosol of host cells, where it serves as a hub protein to disrupt multiple cellular signaling pathways. CagA, when phosphorylated by Src family kinases, can engage with SH2 domain-containing proteins, which are mostly effectors in the mitogenic signaling pathway [79, 80]. Concurrently, unphosphorylated CagA interacts with E-cadherin to disassociate the E-cadherin/β-catenin complex and activate β-catenin-dependent expression of pro-oncogenic genes [81]. In addition to triggering oncogenic signals, CagA also elicits genetic instability, thereby exacerbating the potential for oncogenic transformation. The CagA-mediated sequestration of the polarity-regulating kinase PAR1b, initially known for its disruption of cellular polarity via tight junction perturbation [82], has recently been elucidated to play a role in inhibiting BRCA1 phosphorylation. This inhibition results in the confinement of BRCA1 to the cytoplasm, called BRCAness, where it fails to perform DNA repair in the nucleus, thereby inducing DNA double-strand breaks (DSBs) and genetic instability. The PAR1b kinase inhibited by CagA also activates Hippo signaling, thereby providing DNA-damaged cells with a means to escape apoptosis and facilitating the repair of DSBs via mutagenic repair mechanisms. As the cells with BRCAness expand, the pro-oncogenic role of CagA is eventually replaced by genomic instability which gives rise to cells with cancer predisposition phenotype independent of CagA. Consequently, the neoplastic phenotype no longer relies on the presence of CagA [83].

Moreover, *H. pylori* seems to assume a pioneering role by establishing a low-acid environment conducive to the colonization of potential pathogenic microorganisms in the stomach. For instance, a longitudinal study showed that patients with successful *H. pylori *eradication but still with persistent inflammation exhibit an increase in oral bacteria and pathogenic bacterial load [70]. The mechanisms underlying *H. pylori*-induced hypochlorhydria were partially unveiled. During acute *H. pylori *infection, T4SS protein CagL plays a dual role: it facilitates the intracellular delivery of CagA by binding to α5β1 integrins and also influences the dissociation of the metalloenzyme ADAM17 from α5β1 integrins. CagL stimulates ADAM17 activation, initiating ADAM17-triggered NF-κB-mediated suppression of H,K-ATPase α subunit (HKα) [84]. Besides, pro-inflammatory cytokines IL-1ß and TNF-α released by immune cells elicited by *H. pylori *infection are also robust inhibitors on the gastric acid secretion of parietal cells [85].

The “hit and run” theory was initially introduced by Skinner et al. to elucidate how viruses contribute to carcinogenesis by promoting the accumulation of mutations and inducing genomic instability until the virus is no longer necessary for tumor maintenance [86]. Consistently, genetic instability and neutralized PH in the stomach caused by *H. pylori* infection induces the accumulation of oncogenic insults including driver mutations acquisition in cancer-predisposing cells and pathogenic bacteria colonization in the stomach, which collectively contribute to the establishment and persistence of a neoplastic phenotype. And the actions of *H. pylori *successively fade in malignancy maintenance [87]. Aforementioned longitudinal study reported that eradicating *H. pylori *is insufficient to completely reverse the progression of IM [70]. Moreover, a study incorporating 9 gastritis, 7 IM and 11 GC subjects showed a decrease in *H. pylori *abundance in GC while no significant differences in the abundance of other dominant species between cancer and non-cancer group [55]. Similar conclusions were found in a Portuguese study (54 GC and 81 CG) in which *H. pylori *load reduced with increased abundance of intestinal commensal in GC versus chronic gastritis [57]. Based on a pathology review conducted by Stewart et al., the prevalence of *H. pylori* was found to be as high as 90% in cases of active gastritis, but it decreased to a range of 30% to 72% in individuals with atrophic gastritis and to approximately 30% to 35% in those with intestinal metaplasia (IM) and dysplasia. Notably, only 24.6% of GC patients had detectable *H. pylori* [3]. The “hit and run” model of *H. pylori* was initially introduced and documented by Hatakeyama. On the one hand, it highlights the action of CagA in cell reprogramming by facilitating genetic and epigenetic alternation, on the other hand, elevated genetic instability evokes host cell protection mechanisms such as cell senescence and apoptosis, impeding the role of CagA in transformed cells, ultimately resulting in the diminishing influence of CagA over time. Likewise, in the context of reduced gastric acid secretion and the absence of *H. pylori,* invasive microbiota successfully colonizes the stomach, initiating a progressive replacement of *H. pylori* by other microbial species that eventually come to dominate the gastric environment [87].

#### The interplay between *H. pylori *and non-*H. pylori* microbiota

The presence of *H. pylori* influences the microecological landscape in the stomach. In turn, gastric microbiota can also modulate the outcome of *H. pylori* infection. This interplay between *H. pylori* and the microbiota throughout Correa's cascade is potentially involved in GC development.

*H. pylori *demonstrates a strong competitive advantage, rendering it the predominant microorganism in the gastric microbiota, constituting 40%–90% of the total gastric microbiota composition [30, 38, 57, 88]. The presence of *H. pylori *is inversely correlated with the alpha diversity of gastric bacteria [58, 88–90]. While the impact of *H. pylori* on alpha diversity can be reversible after the removal of *H. pylori* [70, 91]. Evidence from beta diversity analysis suggested that the presence of *H. pylori *infection leads to significant modifications to microbial composition in the stomach [89, 92]. Individuals infected with *H. pylori* exhibit a predominant composition of the same phyla as those without *H. pylori,* but with low levels of Actinobacteria, Bacteroidetes, and the overrepresentation of Proteobacteria, probably attributed to the influence of *H. pylori* [93]. Co-occurring and co-excluding networks showed the potential interplay between *H. pylori* and other gastric microbes. In SG, *H. pylori* manifests a pattern of co-exclusion with *Methylobacillus* and co-occurrence with *Arthrobacter*. Within the stomach of subjects with IM, *H. pylori* demonstrates co-exclusion with various members of the Firmicutes phylum, such as *Ruminococcus*, *Bacillales*, *SMB53* and *Lactobacillus*, while concurrently displaying co-occurrence with *Prevotella*, *Moryella* and *H. ganmani* [53]. Among GC patients, a notable negative correlation persists between *Lactobacillus* and *Helicobacter* [23].

A study employing germ-free insulin-gastrin (INS-GAS) transgenic mice illuminated the crucial role of gastric microbiota in *H. pylori*-driven Correa's cascade progression. *H. pylori* infection in germ-free mice induced milder lesions and delayed progression to gastric intraepithelial neoplasia (GIN), in comparison to *H. pylori*-infected specific pathogen-free (SPF) INS-GAS mice [94]. Remarkably, it has been documented that gastric colonization involving *H. pylori*, along with a consortium of commensal microbiota encompassing species such as *Clostridium* spp., *Bacteroides* spp., and *Lactobacillus murinus*, led to the development of GC in INS-GAS mice to a degree on par with that observed in mice harboring a diverse microbiota. Moreover, the colonization of the stomachs of INS-GAS mice with complex microbiota even reduced the level of gastric *H. pylori* colonization. These findings indicate that the effective colonization with specific commensals seems to exert a greater influence than the overall microbial diversity in the context of *H. pylori*-associated GC [94, 95].

Several gastric microbiota with potential modulatory effects on the severity and outcomes of *H. pylori *infection have been identified [96–101]. For instance, *Staphylococcus epidermidis* and *Streptococcus salivarius*, both urease-positive bacteria, were isolated from individuals in low- and high-cancer-risk regions of Colombia respectively. Co-infection experiments in germ-free mice showed that neither bacterial strain significantly altered *H. pylori* abundance. However, a more severe pathological lesion was found in *H. pylori-S. salivarius* dual infection while *H. pylori-S. epidermidis* co-infection showed alleviation of tissue injury and inhibited gene expression of pro-inflammatory cytokines including IL-22, IL-17A, IL-1β, IFN-γ and TNF-α in contrast to *H. pylori *mono-infection [96].

Moreover, *Weizmannia coagulans* (BCF-01), a strain isolated from the healthy gastric mucosa, displayed strong anti-*H. pylori* activity. This strain could significantly alleviate *H. pylori*-triggered gastric dysbiosis and reduce inflammation following *H. pylori *infection. Mechanistically, it enhanced the expression of mucosal barrier proteins and inhibited the TLR4-NFκB-mediated pyroptotic pathway in macrophages [102]. Similarly, *Lactobacillus gasseri* (Kx110A1), another strain isolated from gastric mucosa, effectively suppressed the synthesis of TNF and IL-6 in macrophages upon *H. pylori *infection through downregulation of ADAM17 [98]. Also *Lactobacillus acidophilus* and *Lactiplantibacillus plantarum,* both indigenous gastric strains, attenuated mucosal inflammation in murine gastritis afflicted with *H. pylori* [99]. Furthermore, *Streptococcus mitis,* commonly found in the human stomach, can inhibit the growth of *H. pylori* and prompt its transformation into a coccoid form when co-cultivated with *H. pylori* [100, 101]. Variations in the prevalence of these bacteria interacting with *H. pylori* within the gastric microbial community may underlie the disparate susceptibilities of individuals to *H. pylori* infection and the development of *H. pylori*-propelled GC.

#### The exploration of mechanisms involving non-*H. pylori* microbiota in gastric carcinogenesis

A recent study by Kwon et al. demonstrated the successful reproduction of premalignant lesions in the stomach by transplanting the gastric microbiota from IM and GC patients to germ-free mice, even in the absence of *H. pylori*. This discovery underscores the contribution of gastric dysbiosis beyond the involvement of *H. pylori *to the induction of GC [103]. While a definitive mechanistic elucidation remains insufficient, numerous investigations have indicated the potential implication of specific bacterial species in GC development.

To begin with, *Streptococcus anginosus* (*S. anginosus*)*,*a species originating from the oral cavity and known for its marked resilience in acidic conditions (pH 3–5), has shown significant overabundance in the gastric mucosa of GC patients across various human cohorts [53, 58, 104]. Administration of *S. anginosus* via oral gavage in conventional and germ-free mice induced progressive precancerous lesions, including gastritis, parietal cell atrophy, mucinous metaplasia, and dysplasia. Further, *S. anginosus* in carcinogen-induced or allograft GC models expedited tumor progression by disrupting gastric barrier function, stimulating cell proliferation and preventing apoptosis. Notably, the influence of *S. anginosus* on gastric carcinogenesis appeared to be species-autonomous, which relied on the interaction between a bacterial lipoprotein, TMPC, and Annexin A2 (ANXA2) receptor on gastric epithelial cells. This interaction instigated oncogenic MAPK signaling cascades, as evidenced by the phosphorylation of ERK and JNK, as well as activation of downstream oncogenic targets within gastric epithelial cells following *S. anginosus *infection [105].

Another frequently cited candidate associated with GC, showing significant overabundance in the gastric microbiota of GC patients, is *Lactobacillus *species [8, 22, 23, 54, 55, 57, 61, 62]. Some hypotheses suggested that *Lactobacillus *spp. may contribute to GC development by producing lactic acid, which could serve as an energy source for tumor cells, promote reactive oxygen species (ROS) production, and contribute to tumor angiogenesis [67, 106, 107]. Nevertheless, the current body of proofs falls short of providing definitive support for these hypotheses. A converse viewpoint has been proposed by several studies, suggesting that *Lactobacillus* spp. could play an anti-tumor role in GC. This includes their potential to activate the intrinsic apoptotic pathway in cancer cells [108], prevent *H. pylori *infection [109, 110], and regulate gastric dysbiosis [111]. Furthermore, recent observations presented an alternative perspective indicating that the overgrowth of *Lactobacillus* spp*. *may be a consequence rather than a cause of gastric carcinogenesis [112, 113]. Jin et al. conducted long-term treatment of INS-GAS mice with deoxycholic acid (DCA), a secondary bile acid that increases from chronic gastritis to intestinal metaplasia. DCA treatment accelerated the progression to IM and enriched the population of the *Lactobacillus *genus [113]. In light of these complexities, further scrutiny is indispensable to elucidate the multifaceted role of *Lactobacillus* spp. in GC.

Additionally, lipopolysaccharide (LPS)-producing bacteria were found to be present at the high relative abundance in the gastric microenvironment of patients suffering from bile reflux gastritis (BRG) and GC. Among these bacteria, an oral-derived pathogen, *Prevotella melaninogenica* (*P. melaninogenica*) was found to be prominently elevated [58, 114, 115]. The abundance of LPS-producing bacteria exhibited a positive correlation with taurodeoxycholic acid (TDCA), a secondary bile acid that can stimulate the growth of gastric epithelial cells by triggering a pro-inflammatory IL-6/JAK1/STAT3 signaling cascade. The gavage instillation of TDCA, LPS, and *P. melaninogenica* in mice induced gastric inflammation and pre-neoplastic lesions, indicating the potential involvement of LPS-producing microbes in the pathogenesis of GC [114]. Therefore, targeting these microbes through alternative interventions may hold promise as a preventive measure against BRG-associated GC.

Microbial species participating in the metabolism of nitrate and nitrite have been implicated in GC. Phylogenetic Investigation of Communities by Reconstruction of Unobserved States (PICRUSt) analysis from multiple studies unveiled distinctive microbial functional characteristics in GC that are linked to heightened reduction reactions, converting nitrate to nitrite, a precursor of N-nitroso compounds (NOC) [57, 61, 63]. Indeed, bacteria possessing nitrate reductase enzymes such as *Clostridium*, *Lactobacillus*, and *Neisseria *were found to be significantly overrepresented in GC patients [23, 55, 57, 91]. Conversely, *Nitrospira*, a bacterium that oxidizes nitrite to nitrate, were found to progressively decrease in individuals with intraepithelial neoplasia (IN), IM and GC. This decrease may indicate the accumulation of nitrite during the progression of neoplastic lesions [52]. The buildup of intragastric nitrite and NOC facilitates malignant transformation by initiating DNA damage through the methylation of purine and guanine with electrophilic species, thereby increasing mutagenicity [116, 117].

A novel investigation unveiled distinctive mucin-microbiome profiles in association with clinical outcomes in GC. The results indicated that tumors displaying an intestinal mucin phenotype with upregulated MUC13 expression are associated with an unfavorable prognosis, whereas tumors characterized by dominant expression of MUC5AC or MUC6 exhibit a more encouraging outcome. The presence of specific oral or intestinal microbes was discovered to be dependent on the mucin phenotype. Notably, oral taxa such as *Neisseria*, *Prevotella*, and *Veillonella *showed a significant association with MUC13 overexpression [118]. In prior studies, MUC13 overexpression was documented in GC [119], and this dysregulated MUC13 signaling was shown to confer protection against apoptosis in colorectal cancer (CRC) cells through the activation of the NF-κB pathway [120], as well as to facilitate the progression of intrahepatic cholangiocarcinoma by activating the EGFR/PI3K/AKT pathways [121]. Nevertheless, its specific involvement in GC has yet to be thoroughly investigated. Whether such interactions between mucin and the microbiome causally contribute to gastric carcinogenesis remains an intriguing area for further exploration.

Microbiota-mediated epigenetic regulation represents a novel mechanism by which gastric bacteria may contribute to carcinogenesis [122]. For instance, tumors harboring *F. nucleatum *exhibited a trend towards decreased DNA methylation of the long interspersed nuclear elements 1 (LINE1) [71]. *Kytococcus sedentarius, Actinomyces oris* and *Staphylococcus saccharolyticus* were implicated in regulating DNA methylation of immune-related genes in gastric adenocarcinoma, which consequently promoted distal metastasis in GC. In an in vitro experiment, the co-culture of GC cells with *Staphylococcus saccharolyticus *was observed to enhance the proliferation and clonogenicity of GC cells by inducing loss of ZNF215 methylation imprinting [123].

Several microorganisms implicated in other cancers were observed to be also enriched in GC [23, 55, 60, 91]. For instance, *F. nucleatum*, a periodontal commensal, was reported to be involved in the carcinogenesis of CRC [124]. Two vitro studies provided preliminary insights into the potential role of *F. nucleatum* in the development of GC. Hsieh et al. revealed that *F. nucleatum *colonization results in the dysregulation of actin cytoskeletal dynamics, which in turn is likely to alter the motility of GC cells [72]. In another study, it was shown that *F. nucleatum *is capable of inducing the overexpression of exosomal HOTTIP from GC cells, which subsequently accelerates the progression of GC through the activation of the miR-885-3p/EphB2/PI3K/AKT signaling cascade [125]. However, the impact of *F. nucleatum *on GC lacks validation from in vivo animal studies, and the molecular mechanisms underlying this effect are not fully understood. In CRC, the carcinogenic effect is primarily mediated by its virulence factors [8, 64, 126, 127]. Fap2 lectin ensures the localization and enrichment of *F.nucleatum *in cancerous sites [128, 129]. And the interaction of FadA adhesin and E-cadherin of cancer cells over-activate the β-catenin/Wnt signaling pathway [130]. Additionally, LPS of *F.nucleatum *recognized by the Toll-Like Receptor 4/Myeloid Differentiation Primary Response 88 (TLR4/MYD88) system induces NF-κB signaling pathway activation [131]. Notably, sustained activation and abnormal modulation of the NF-κB signaling pathway play a crucial role in the maintenance of stem-cell-like prosperity of GC cells [131–137]. Except for *F.nucleatum*, *H. pylori* has been demonstrated to stimulate NF-κB signaling pathway activation via its distinctive effector molecule, ADP-glycerol-β-D-mannoheptulose [138, 139], *Peptostreptococcus anaerobiu*s and *Bacteroides fragilis *which were observed to increase in GC, can activate NF-κB signaling pathway via inducing intracellular synthesis of ROS and IL-17 respectively in CRC [140, 141]. However, it remains unclear whether these mechanisms of the bacteria are replicated in the acidic environment of the stomach.

### Non-bacterial component in gastric cancer

Although the majority of investigations into the connection between the microbiome and GC have primarily focused on bacterial components, a limited number of studies adopting a pan-biological perspective have yielded insights into the tumorigenic potential of non-bacterial elements, conferring a more comprehensive depiction of the microbiome ecosystem within the context of cancer [142–144].

One published by Zhong et al. in 2021 initially characterized fungal features of GC by applying internal transcribed spacer (ITS) rDNA sequencing on 45 gastric cancerous biopsies and peritumor normal biopsies. *Candida* and *Alternaria* were detected as representatives of fungi in cancerous tissue whereas *Saitozyma* and *Thermonyces* were observed to decrease in GC. Although the causal role of fungus on gastric carcinogenesis has not been proved, they highlighted the role of *Candida albicans*as an effective biomarker to distinguish GC tissue from control [143]. *Candida albicans *is a common fungus in human microbiota that normally colonizes but can cause severe issues in immunocompromised patients [145]. Recently bioinformatics analysis of mycobiome performed by Dohlman et al. demonstrated the unique enrichment of *Candida* in GC tissues. Concerning endosymbiotic crosstalk, authors conducted trans-kingdom network analysis and identified *Candida* as “Keystone taxa” in cancer which is co-abundant with a cluster of tumor-specific microbiota including *Streptococcus*, *Clostridium*, and *Lactobacillus* in GC. Moreover, they revealed *Candida*-corresponding gene expression patterns in GC, where pro-inflammatory cytokines such as IL-8, IL-1β, and IL-6 were mainly upregulated, indicating the involvement of *Candida* in inflammation in GC. However, it is still elusive whether overrepresented *Candida *is a cause or secondary to the phenotype of GC [144].

The majority of Mycoplasma implicated in GC are *Mycoplasma hyorhinis*(*M. hyorhinis*), a recognized pathogen responsible for various swine diseases [127, 146]. Experimental studies involving co-culturing *M. hyorhinis* with host cells have shed light on its impact on GC cell lines. Lipoprotein P37, expressed on the membrane of *M. hyorhinis*, serves as the main virulence factor, significantly enhancing the invasiveness of GC cells. This enhancement is strongly correlated with the activation of the EGFR-dependent NF-κB signaling pathway and the activation of matrix metalloproteinase-2(MMP-2) [147, 148]. Besides, Lipid-associated membrane protein(LAMP) of *M.hyorhin, *recognized by TLR2, induces the monocyte-derived IL-1ß secretion in an NLRP3 inflammasome-mediated manner to enhance cell migration [149]. Furthermore, *M.hyorhin*-specific methyltransferases selectively act on CG and GATC sites may lead to pro-carcinogenic epigenetic alternation by silencing tumor-suppressing genes [150].

## Microbial involvement in immunosurveillance of gastric cancer

**Fig.3:**
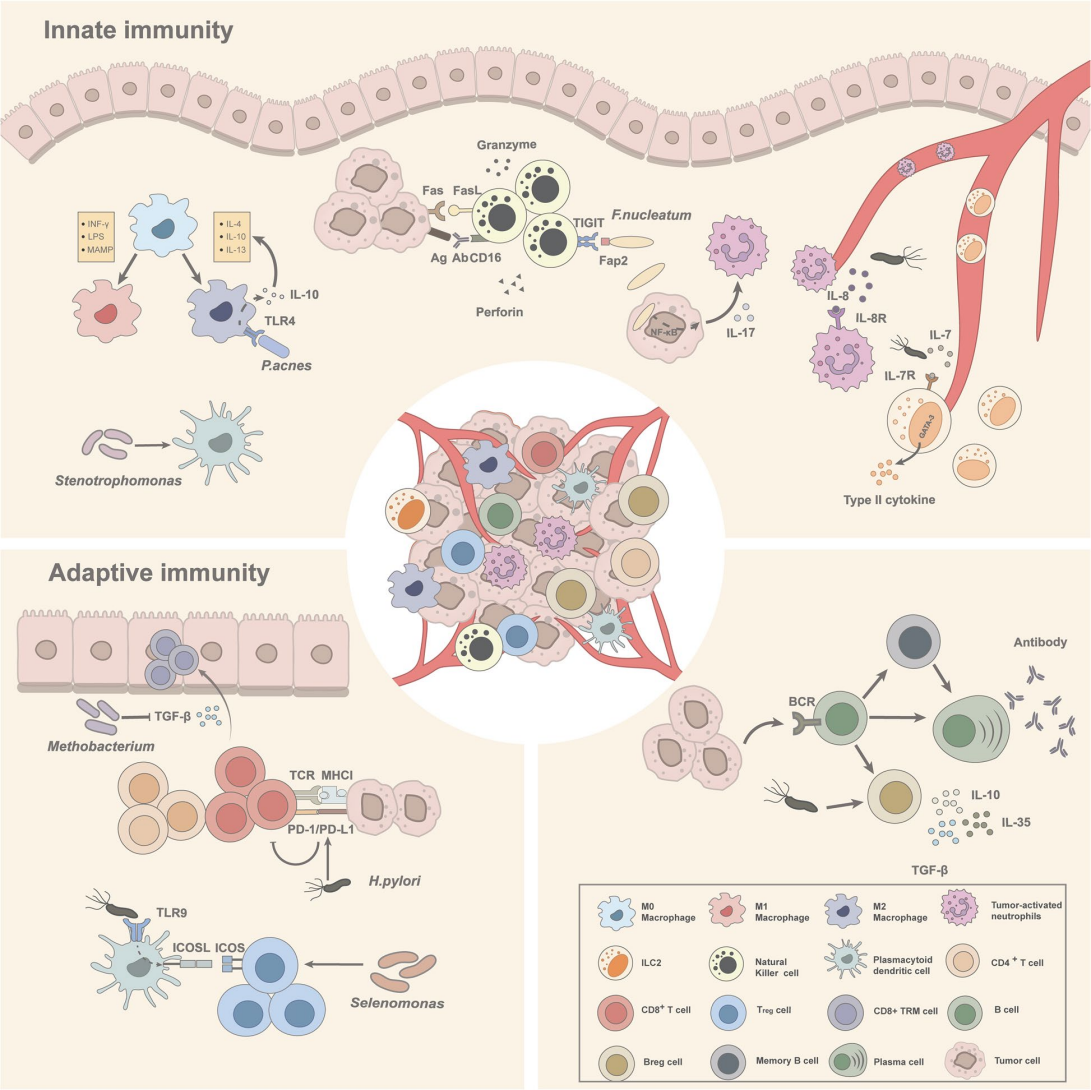
The impact of gastric microbiota on the modulation of both innate and adaptive immunity within the tumor microenvironment of GC. Innate immunity: Macrophage: *Propionibacterium acnes* drives the polarization of macrophages into the M2 phenotype by activating TLR4, leading to the secretion of IL-10. pDCs: *Stenotrophomonas* demonstrates a favorable correlation with the infiltration frequency of pDCs. NK cells: *Fusobacterium nucleatum* hinders the tumoricidal activity of NK cells by engaging in binding interactions with the inhibitory receptor TIGIT via its Fap2 lectin. ILC2s: *H. pylori* induces an upregulation in the expression of IL-7R on ILC2s and increases the levels of IL-7 in the gastric environment, thereby promoting the enhanced recruitment of ILC2s. Tumor-activated neutrophils: *H. pylori* infection induces an elevation in IL-8 secretion, which subsequently attracts the infiltration of neutrophils. *F. nucleatum* residing in GC cells activates the NF-κB /IL-17 axis, promoting neutrophils recruitment. Adaptive immunity: CD8 + TRM cells: *Methobacterium* exerts inhibitory effects on TGF-β secretion, leading to a disruption in the residence of CD8 + TRM cells within the gastric epithelium. Effector T cells: *H. pylori* impedes the recognition of tumor cells by effector T cells by upregulating the expression of PD-L1 within the tumor microenvironment. Treg cells: *Selenomonas* demonstrates a positive correlation with the frequency of infiltration of Treg cells. *H. pylori* induces the activation of TLR9 on pDCs, which may contribute to the overexpression of ICOSL and subsequently facilitate the recruitment of Treg cells. Breg cell: *H. pylori* is associated with the elevation of Breg cells infiltration in the gastric microenvironment

In a state of homeostasis, the persistent mutualistic symbiosis between the host and commensal flora not only serves to safeguard the microbial ecosystem but also plays a crucial role in preserving immunological equilibrium. Nonetheless, when the intricate interplay goes awry due to dysbiosis or disruptions in the host system, opportunistic pathogens can seize the opportunity to initiate pathological processes. In this scenario, pathogen-associated molecular patterns (PAMPs), such as flagellin, LPS, peptidoglycan, and formylated peptides recognized by pathogen recognition receptors (PRRs) of host cells may initiate an aberrant immune response. Similarly, within the realm of cancer, the presence of tumor-enriched pathogens harbors the capacity to disrupt the balance between immune defenses and tumor evasion, ultimately facilitating the progression of the disease [75] (Fig. 3).

### Potential role of gastric microbes on the innate immune system in gastric cancer

Innate immunity encompasses a diverse array of myeloid or lymphoid lineages-derived immune cells, cytokines and complement cascades, all working in concert to coordinate immune balance. The innate immune cells do not possess an intrinsic predisposition to either favor or impede tumors. Instead, their phenotype and function exhibit plasticity, influenced by contextual cues within specialized niches and the signaling molecules they encounter. Among these, the microbial pattern acts as a key component that significantly shapes the plasticity, recruitment, activity, and overall functionality of the innate immune system. In this regard, we present pivotal findings that shed light on the remodeling of innate immune cells by the microbiota within the TME of GC, and how these interactions exert profound effects on the course of cancer.

Macrophages are a significant component of the leukocytic infiltrate and exhibit phenotypic plasticity in GC. The M1 phenotype, triggered by IFN-γ and microbes-derived molecules, has anti-tumor activities. Conversely, the M2 phenotype, induced by IL-4, IL-10, and IL-13 cytokines, exerts an immunosuppressive effect, encouraging the immune escape of cancer cells [151]. A recent study revealed that *P. acnes*, a skin pathogen prevalent in GC, can drive the polarization of macrophages toward the M2 phenotype, facilitating GC cell migration in vitro. Pre-exposure of macrophages to *P. acnes* induces the expression of TLR4 on their surface. This, in turn, initiates an interaction between TLR4 and *P. acnes*, activating the downstream PI3K/AKT signaling pathway, which upregulates IL-10 expression, a canonical stimulus for promoting M2 polarization. Furthermore, co-localization analysis of M2 and TLR4 in GC tissue samples observed sustained expression of TLR4 in the majority of M2 macrophages. This observation implies that the overrepresentation of *P. acnes *within TME may be involved in a positive-feedback loop for M2 polarization, as self-secreted IL-10 further enhances the M2 phenotype [152].

Group 2 innate lymphoid cells (ILC2s), the principal subset of innate lymphoid cells in the stomach, respond to tissue injury and bacterial exposure to expand with the production of type II cytokines (IL-4, IL-5, IL-9, and IL-13) [153]. The mutualistic relationship between ILC2s cells and the microbiota is implicated in gastric homeostasis maintenance. ILC2s-derived IL-5 efficiently stimulates B cells to secrete IgA, which coats gastric bacteria for elimination. Reciprocally commensal bacteria such as *Bacteroidales Family S24-7 *actively participate in the preservation of the abundance and functionality of ILC2s within the stomach [154]. However, in TME, the microbiota-ILC2 interaction undergoes dysregulation, leading to a pronounced skew favoring a pro-tumor activity. The ILC2s in the peripheral blood of GC patients were found to exhibit an immunosuppressive phenotype [155]. And Li et al. reported that *H. pylori *infection induces the overexpression of transcription factor GATA-3 in lymphocytes in GC, contributing to the reprogramming of immune responses towards a dominant type 2 immunity, which is predominantly mediated by immunosuppressive ILC2s [156]. Simultaneously, *H. pylori *infection elicits an upregulation of the surface expression of interleukin-7 receptor (IL-7R) on ILC2s and induces an elevation in the gastric levels of IL-7, which synergistically enhances the recruitment and propagation of ILC2s in the stomach, potentially exacerbating the immunosuppressive nature of the TME [154, 157].

Tumor-activated neutrophils (TANs), a subset of granulocytes, constitute an immune-inhibitory milieu and are also recruited in response to microbial influences in TME [158]. TANs orchestrate a multifaceted role in promoting tumorigenesis by producing pro-angiogenic factors [159], enhancing the metastatic propensity of cancer cells [160] and suppressing anti-tumor immune responses [161]. Their mobilization in GC can be mediated through chemotaxis along the CXCL6/CXCL8-CXCR1 pathway [162]. Studies highlighted that *H. pylori*-induced upregulation of hepatocyte growth factor and IL-8 (CXCL8) triggers the infiltration of neutrophils in chronic gastritis and GC [163]. Moreover, IL-17, a cytokine abundant within the mucosa, was also demonstrated to engage in crosstalk with cancer cells, coordinating the recruitment of neutrophils [164, 165]. The intracellular inhabitation of *F. nucleatum *in GC cells was observed to trigger the NF-κB /IL-17 signaling axis, leading to neutrophil recruitment and facilitating immune evasion by tumor cells [166].

In addition, several bacteria, despite their roles remaining partially understood, were identified in association with specific immune cell populations within TME of GC. For instance, an opportunistic pathogen, *Stenotrophomonas *was found to exhibit a positive correlation with the infiltration frequency of BDCA2 + plasmacytoid dendritic cells (pDCs) in GC tissues [167]. These pDCs, existing in an immature state, are known to positively regulate the activity of regulatory T cells (Treg), thereby promoting the suppression of anti-tumor immunosurveillance [168, 169]. Likewise, *Fusobacterium *sp. infection showed a positive association with the lymphocyte influx in GC [126]. The direct immunosuppressive impact of *F. nucleatum *on human Natural Killer (NK) cells were elucidated in CRC, involving its interaction with the inhibitory receptor TIGIT via the virulence protein Fap2 [170]. However, the applicability of this immunosuppressive mechanism in GC requires further investigations to validate.

### Potential role of gastric microbes on the adaptive immune system in gastric cancer

The adaptive immune system, composed of T-cell-mediated and B-cell-mediated immunity, plays an important role in the surveillance and defense against neoplastic growth. The intricate interplay between the gastric microbiota and the adaptive immune system emerges as a significant determinant in shaping the anti-tumor immune response, thus offering the potential to modulate the dynamic process of GC development and progression [22, 65, 126].

The plasticity of T cells is dependent on various stimuli and signals encountered during lineage differentiation and activation [171]. Studies illustrated the impact of gastric microbiota on the balances of effector and Tregs as well as the regulation of co-stimulatory and co-inhibitory signal transmission [75, 172, 173]. For instance, the overabundance of *Methylobacterium *in GC tissue, highly associated with unfavorable prognostic outcomes in GC patients, was demonstrated to advance GC progression by exhausting CD8 + tissue-resident memory T cells (TRMs) in TME [65, 66]. TRMs, distinguished by their elevated expression of immune checkpoint molecules and effector proteins, serve as a reserve force in anti-tumor immunity and was recognized as a favorable prognostic indicator linked to prolonged patient survival in prior studies [174]. Animal experiments unveiled *Methylobacterium*'s capacity to diminish CD8 + TRMs by downregulating TGFß. This is significant since TGFß plays a pivotal role in the induction of CD103 expression, an essential prerequisite for the TRMs generation and enduring residence within the epithelial tissue [65].

Another bacterium that hampers T cell-mediated anti-tumor immunity is *H. pylori.* Aydın et al. reported an upregulation of immune-checkpoint inhibitor proteins (PD-1/PD-L1) in *H. pylori*-positive GC samples [175]. Experimental studies unveiled a putative mechanism underlying *H. pylori*-mediated PD-L1 expression. Using GC organoids, it was discovered that CagA from *H. pylori *stimulates increased secretion of the signaling molecule Shh, thereby augmenting the Hedgehog signaling pathway. This intricate cascade of events has the potential to modulate PD-L1 expression by regulating the mTOR pathway [176, 177].

Tregs emerge as pivotal orchestrators of the balance of immune tolerance and immune activation in homeostasis. However, within the complex landscape of cancer, a perturbation unfolds, where with the dominance of Treg cells surpasses that of effector T cells, which skews TME towards a state of immunosuppression. This dysregulation, in turn, allows cancer cells to evade vigilant immune surveillance [178]. Foxp3 + Tregs were reported to be over-represented in gastric tumoral and peritumoral samples compared with normal tissue. *Selenomonas*, a typical gingival bacterium, enriched in GC, exhibited a positive correlation with the elevated level of the Foxp3 + Tregs as shown in Pearson's correlation analysis. This suggests its potential contribution to Tregs accumulation [22, 167]. Moreover, a strong association was observed between *H. pylori *infection and the presence of ICOS + Tregs, of which higher level was linked to an unfavorable prognosis in late-stage GC [179]. Within the realm of co-stimulatory receptors belonging to the CD28 family, ICOS stands out as a distinct subtype, characterized by its unique ability to enhance the efficiency of producing immunosuppressive cytokines, including IL-10 and TGF-β. The presence of *H. pylori *in the gastric environment sets off a chain of events, activating TLR9 and instigating the induction of ICOS ligands on pDCs. This process serves as a compelling signal summoning ICOS + Tregs to infiltrate the TME of GC [180].

It was established that B lymphocytes act as anti-tumor immune agents by producing counterpart antibodies [181]. The immunoglobulin pool and repertoire can be influenced by microbial exposure, which may affect B cell recognition and function of tumor cells [182]. A subtype of B cell identified as B regulatory cells (Breg) exhibit a series of immunosuppressive features including synthesis of IL-10, IL-35 and TGF-β, counteracting the activity of effector T cells and NK cells, which are implicated in the immune evasion mechanisms employed by tumor cells [183, 184]. Evidence showed that the density of IL-10-producing B cells was elevated in the presence of *H. pylori* [185]. However, how Bregs respond to *H. pylori* and other microbes is still less well-studied.

## The impact of microbiota on cancer immunotherapy

Immunotherapy that aims to boost the body's immune system to target and eliminate cancer cells has been well-established in clinics. However, it only works well for a small subset of cancer patients, underscoring substantial challenges in achieving favorable outcomes, as a significant proportion fails to mount an adequate response or develops treatment resistance [186]. Robust evidence highlighted the regulation of the microbiome on immune surveillance, inspiring the investigation into the impact and application of microbes on immunotherapy [75, 175, 187]. Given considerable inter-individual variability in the microbiome, the patient's distinctive microbial profile may serve as a pivotal factor in predicting and optimizing the therapeutic response to immunotherapy.

### Gut microbiota and immunotherapy

The lion's share of experimental investigations exploring microbiota's impact on immunotherapy has revolved around gut microbiota, revealing several microbial taxa associated with immunotherapy efficacy [188–192]. For instance, *Bifidobacterium* genus, *Bacteroides fragilis*, *Faecalibacterium* spp., *Akkermansia muciniphila, Ruminococcaceae* and *Lachnospiraceae *were frequently identified as “favorable” bacteria to improve immune checkpoint inhibitors (ICIs) in multiple solid tumors by potentiating anti-tumor immunity [188, 193–198], while the high abundance of *Bacteroidales *was reported to be associated with poor response and weakened tumor-specific immune response [192, 195]. In GC patients, Peng et al. observed an increase in microbial taxa associated with favorable responses to ICIs, including *Prevotealla*, *Bifidobacterium*, *Bacteroides*, *Ruminococcaceae*, and *Lachnospiraceae,* whereas non-responders showed a higher abundance of *Megamonas*, *Butyricimonas*, *Lachnospiraceae_UCG-001*, and *Agathobacter* [199]. This study exclusively concentrated on gut microbial alternation, even though the modulation of the local immune response in the stomach by the gastric microbiota is anticipated to have a potential influence on the efficacy of ICI.

### The impact of *H. pylori* infection and eradication on Immunotherapy

Recently, the impact of *H. pylori* in gastric microbiota on immunotherapy is increasingly becoming a topic of great interest. Oster et al. found that the responsiveness of ICIs is hampered by *H. pylori* infection in both MC38 colon adenocarcinoma and B16-OVA melanoma-bearing mice model as well as non-small cell lung cancer (NSCLC) patients. The tumor size in mice with *H. pylori* infection was much larger than those without infection after anti-CTLA4 or anti-PD-L1 treatment. Likewise, following treatment with anti-PD-1, the median survival time of NSCLC patients with *H. pylori* seropositivity was found to be significantly shorter in comparison to their seronegative counterparts. The mechanistic investigation in murine models unveiled that the infection causes the hypoactivation of DCs, ultimately leading to compromised activation and proliferation of tumor-specific CD8 + T cells. Concurrently, a decrease in the abundance of monocyte-derived cells and a noteworthy suppression of genes under the regulation of type I IFN, IFNγ, and IL-6 were detected within the TME of NSCLC patients who exhibited resistance to ICIs therapy and had pre-existing *H. pylori *infection [200]. The other two retrospective cohort studies confirmed the negative impact of *H. pylori* infection on the response to ICIs. Both ICI-treated melanoma and GC patients with *H. pylori* seropositivity experienced lower overall survival rates and worse treatment outcomes than *H. pylori*-seronegative patients [201, 202].

Notably, despite the adverse impact of *H. pylori* on immunotherapy, Oster et al. were unable to restore the efficacy of immunotherapy even after the successful eradication of *H. pylori*. They attributed this outcome to the decrease in immune-boosting bacteria resulting from non-specific antibiotics (ATB) treatment [200]. This finding is consistent with many clinical investigations that demonstrated a correlation between the use of antibiotics and decreased clinical efficacy of ICIs [190, 203–205]. Regarding the taxonomic composition of the gut microbiome post-eradication of *H. pylori*, several studies reported a gut dysbiosis characterized by a reduction in alpha diversity [206, 207] and *Bifidobacterium *abundance [208, 209]. However, longitudinal studies observed that these effects are transient, with a gradual recuperation of alpha diversity over time [210] and even favorable changes in fecal microbiota composition observed in patients, such as a noteworthy enrichment of *Bifidobacterium* and a decrease in *Bacteroidales*, after a period of 6 months post-eradication [91]. Moreover, different time windows of exposure to ATB before ICI initiation may exhibit different therapeutic outcomes. A comprehensive meta-analysis conducted by Lurienne et al. found that ATB exposure within 60 days of ICI administration significantly reduced its effectiveness in NSCLC patients, while exposure more than 90 days prior did not result in worse clinical outcomes [211].

Although the impact of antibiotics in *H. pylori* eradication on immunotherapy needs to be explored in prospective preclinical and clinical studies that include multiple time points, there seems to be agreement that antibiotics lead to transient ICI-unfavorable perturbations of microbial homeostasis, which should be considered in immunotherapy regimens for cancer patients.

### The therapeutic potential of *H. pylori* in immunotherapy

In addition to the immunosuppressive effects of *H. pylori *infection within the TME, multiple researchers have delved into the strategic harnessing of its immunostimulatory property to augment the therapeutic efficacy of immunotherapy [212–215].

Helicobacter pylori neutrophil-activating protein (HP-NAP) is a virulence factor in *H. pylor*i, known for its ability to activate neutrophils and induce reactive oxygen species via TLR2 activation [216]. HP-NAP activates neutrophils and monocytes, promoting IL-12 and IL-23 production, which polarizes a CD4 + T cell response into an anti-tumor Th1 response characterized by increased IFN-γ and TNF-α [217]. It also aids DC maturation and shifts macrophages towards an anti-tumoral phenotype [218]. These potent immunogenic properties make HP-NAP a promising candidate for the development of novel cancer immunotherapeutic applications.

Given these, Codolo et al. conducted the first investigation of the therapeutic potential of HP-NAP in bladder cancer-bearing mice. Peritumoral administration of HP-NAP resulted in a reduction of tumorigenesis, promotion of tumor necrosis, and augmentation of CD4 + and CD8 + cell populations secreting IFN-γ within both tumor sites and associated lymph nodes [219]. In subsequent studies, the efficacy of Hp-NAP in various formulations was extensively explored for its anticancer properties. Hp-NAP recombined with the maltose binding protein of *Escherichia coli*(rMBP-NAP) stimulated TLR2-mediated Th-1-dependent anti-tumor immunity in hepatoma, sarcoma [220], and metastatic lung cancer [221]. Recombinant HP-NAP-embedded Chitosan nanoparticles (Chi-rNap) was reported to reduce breast tumor growth by enhancing cytokine production and shifting immune functionality towards tumor-killing [222]. And the genetic manipulation of oncolytic viruses (OVs) to HP-NAP, harnessing the selective oncolysis of OVs with the immunomodulatory properties of HP-NAP, was demonstrated to amplify anti-tumor immune responses in breast cancer and neuroendocrine tumors [212, 213]. Moreover, a recent study introduced chimeric antigen receptor (CAR) T cell-bearing secretory HP-NAP as a solution for CAR-T's limited effectiveness in solid tumors. CAR(NAP) T cells prompted DC maturation, elicited bystander T-cell responses, and activated cytotoxic CD8 + T cells against multiple tumor antigens, not just the CAR's target. Importantly, CAR(NAP) T cells didn't increase non-specific toxicity or hinder CAR T cell therapy, offering promising clinical potential [214]. These remarkable findings highlight the immense potential of *H. pylori* in revolutionizing immunotherapy, although practical applications in GC are pending.

## Conclusion and prospect

Growing numbers of studies in recent years have uncovered the alternation of gastric microbiota during gastric carcinogenesis, particularly changes in diversity and composition. These changes may serve as promising biomarkers for clinical applications of GC. Additionally, the GC-associated microbial community actively helps tilt TME towards a more immunosuppressive state, contributing to the evasion of cancer cells from immune detection and clearance. Notably, *H. pylori* possesses an intricate immunomodulatory mechanism. On one hand, its immunosuppressive nature poses a detrimental impact on the effectiveness of immunotherapy. On the other hand, the virulence factors of *H. pylori* exhibit remarkable immune-stimulating properties, holding promise as adjunctive mediators for immunotherapy.

However, the area is still in its infancy and the tasks trying to elucidate the underlying mechanism through which gastric microbiota influence the carcinogenic process and mediate immunomodulation are challenging. For instance, past retrospective investigations presented intriguing and contentious evidence for linking gastric microbes and cancer. This presumably reflects the co-evolutionary dynamics of the host's immune system, its symbiotic microbiota, and tumorigenic processes, and such dynamic changes are typically unable to be captured by cross-sectional comparison. Concurrently, the variability in sequencing methodologies presents a significant hurdle in attaining a consensus regarding the landscape of the gastric microbiota. Thus, it is necessary to allocate increased research efforts to this area, including the establishment of multi-center longitudinal cohort studies and the application of standardized sequencing tools with the capacity to dissect the entire metagenome at the strain level (e.g., emerging third-generation sequencing technologies [223] and single-cell microbial sequencing [224]). 

Furthermore, despite the extensive interest in the involvement of non-*H. pylori *gastric microbiota in GC, there is limited evidence regarding their causality with GC and their roles in the TME [173]. In light of this, there is a compelling need for in-depth research to monitor the co-evolutionary dynamics within the gastric microbiota. These researches are aimed at achieving a more comprehensive understanding of its multifaceted role in the tumorigenesis process. It is noteworthy to underscore that this exploratory venture should transcend the bacterial taxonomic level and encompass the full spectrum of microorganisms.

**Fig. 4:**
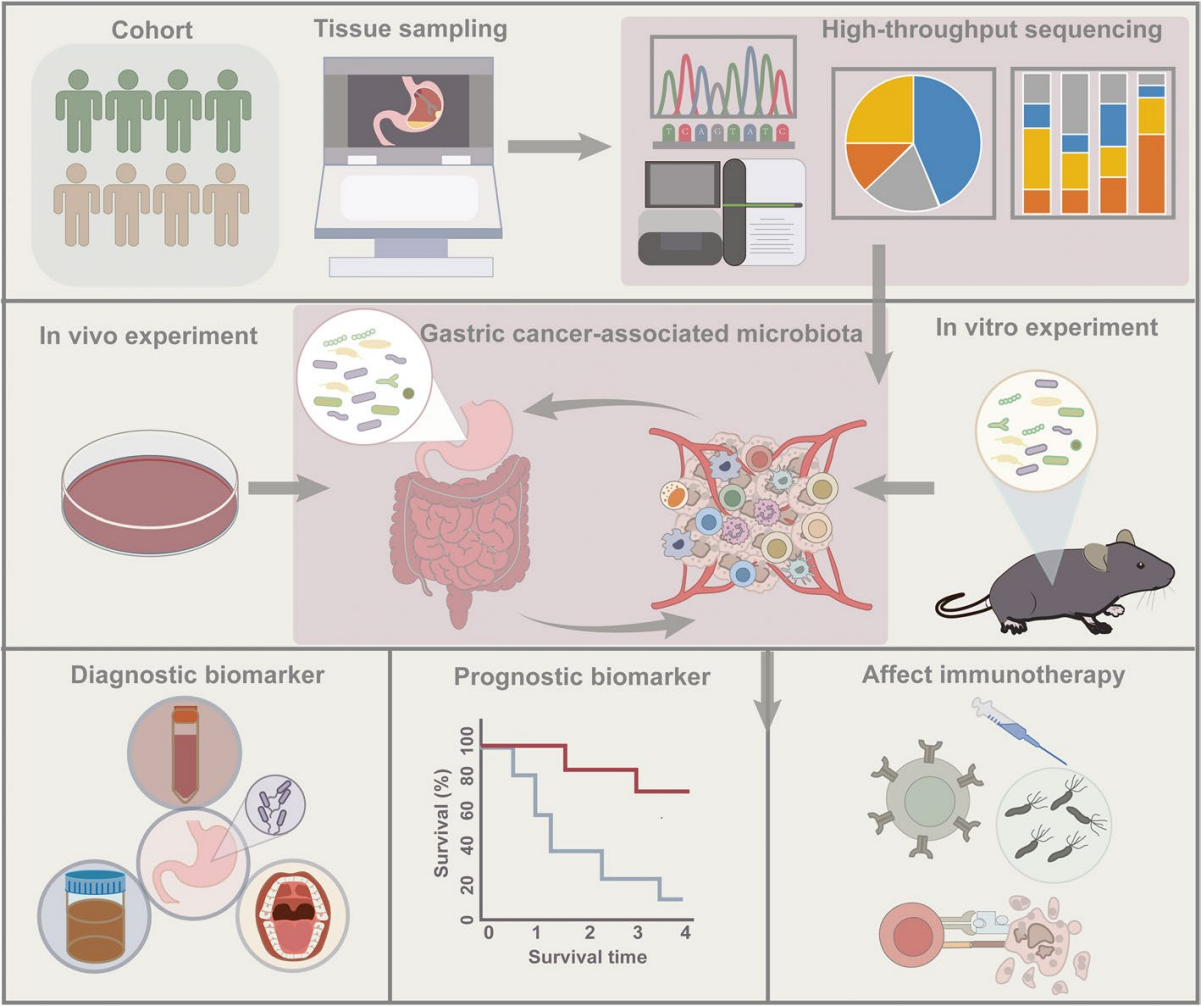
Advancing the Translation of Gastric Microbial Research: from clinic to clinic. The multicenter longitudinal cohort studies employ standardized strategies crafted to enhance the precision of data collection and analysis related to gastric microbiota. By incorporating a diverse range of genetic and non-genetic methodologies and considering potential influencing factors affecting outcomes, while recognizing inter-individual variability, the initiative aims to advance the comprehension of the intricate interplay between the microbiota and host co-evolution. Through the integration of both in vitro and in vivo studies, the research goes beyond mere identification of associations, correlations, and predictions. Instead, it delves into the exploration of mechanistic underpinnings behind causal relationships, providing profound insights at the molecular level. This concerted effort lays the foundation for personalized diagnostics and microbiota-based synergistic therapies in the intervention of gastric cancer

Exploring the clinical translation related to microbiota is a pivotal area of future research. Nevertheless, significant challenges persist in the clinical application of gastric microbiota. The invasive nature of endoscopy poses a substantial impediment to early GC screening, potentially limiting the clinical adoption of gastric mucosal microbiota testing as a viable biomarker. Moreover, current studies investigating the influence of gastric bacteria on cancer prevention and therapeutic response are primarily focused on *H. pylori*, with other strains remaining underexplored. Additionally, the applicability of established microbiota-based therapeutic strategies, including prebiotics, symbiotic microbial consortia, microbiota-derived metabolite therapy, microbiota-targeted interventions, and microbiota engineering, to the gastric microbiota ecosystem remains unclear, particularly given the stomach's unique acidic conditions. Interestingly, despite these challenges, emerging technologies and accumulating evidence indicate some limited progress in this field. For example, the detection of GC-related microbial translocation in the blood [225], oral cavity [56], and feces of GC patients has shown promising predictive significance and offers a non-invasive means for early GC screening [226]. Regarding of microbiota-targed treatment, studies have investigated strategies to target *H. pylori*, such as developing biomaterials that utilize the gastric acid environment as an activation mechanism to selectively eradicate *H. pylori* from the stomach [227, 228], and genetically modifying probiotics to release *H. pylori*-guided antimicrobial peptides [229]. These approaches have the potential to eliminate *H. pylori,* all while avoiding the induction of gastric dysbiosis. However, extensive clinical trials, underpinned by rigorous preclinical experiments, are essential to assess the safety and efficacy of these strategies and enhance our comprehension of GC prevention and treatment through the modulation of gastric microorganisms, extending beyond *H. pylori* (Fig. 4).

Going forward, the personalized treatment of cancer will increasingly integrate the microbiome with genomic, transcriptomic, and metabolomic data in the future. This multi-level and multi-dimensional approach will enable a more comprehensive and nuanced understanding of the interplay between the gastric microbiome and GC, facilitating the development of targeted and effective treatment strategies.

## Data Availability

Not applicable.

## Abbreviations

GC: Gastric cancer
TME: Tumor microenvironment
TGGE: Temperature gradient gel electrophoresis
FISH: Fluorescence in situ hybridization
NGS: Next-generation sequencing
WGCNA: Weighted gene co-expression network analysis
GI: Gastrointestinal
HC: Healthy control
AG: Atrophic gastritis
SG: Superficial gastritis
CAG: Chronic atrophic gastritis
IM: Intestinal metaplasia
GIN: Gastric intraepithelial neoplasia
OTUs: Operational Taxonomic Units
ORs: Odds ratios
WMS: Whole metagenomic shotgun sequencing
EGN: Early gastric neoplasia
NS: Not significant
VacA: Vacuolating cytotoxin A
CagA: Cytotoxin-associated gene A
cagPAI: Cag pathogenicity island
T4SS: Type IV secretion system
SH2: SRC homology 2 domain
PAR1b: Partitioning-defective 1
BRCA1: Breast cancer susceptibility gene 1
DSBs: DNA double-strand breaks
ADAM17: Metalloproteinase domain 17
NF-κB: Nuclear factor-κB
HKα: H,K-ATPase α subunit
IL-1ß: Interleukin-1ß
TNF-α: Tumor Necrosis Factor-α
INS-GAS: Insulin-gastrin
SPF: Specific pathogen-free
ANXA2: Annexin A2
IFN-γ: Interferon-γ
ROS: Reactive oxygen species
DCA: Deoxycholic acid
LPS: Lipopolysaccharide
BRG: Bile reflux gastritis
TDCA: Taurodeoxycholic acid
JAK1: Janus kinase 1
STAT3: Signal transducer and activator of transcription 3
PICRUSt: Phylogenetic investigation of communities by reconstruction of unobserved States
NOC: N-nitroso compounds
MUC: Mucin
LINE1: Long interspersed nuclear elements 1
CRC: Colorectal cancer
HOTTIP: HOXA transcript at the distal tip
TLR: Toll-Like Receptor
MYD88: Myeloid Differentiation Primary Response 88
NOD1: Nucleotide-binding Oligomerization Domain 1
ITS: Internal transcribed spacer
MMP-2: Matrix metalloproteinase-2
LAMP: Lipid-associated membrane protein
NLRP3: Nucleotide-binding oligomerization domain, leucine-rich repeat and pyrin domain-containing protein 3
PAMPs: Pathogen-associated molecular patterns
PRRs: Pathogen recognition receptors
ILC2s: Group 2 innate lymphoid cells
IL-7R: Interleukin-7 receptor
TANs: Tumor-activated neutrophils
pDCs: Plasmacytoid dendritic cells
Treg: Regulatory T cells
NK: Natural Killer
TIGIT: T-cell immunoreceptor with Ig and ITIM domains
TRMs: Tissue-resident memory T cells
PD-1/PD-L1: Programmed Cell Death Protein 1/Programmed Death-Ligand 1
shh: Sonic Hedgehog
ICOS: Inducible T-cell CO-Stimulator
Foxp3: Forkhead Box P3
Breg: B regulatory cells
ICIs: Improve immune checkpoint inhibitors
NSCLC: Non-small cell lung cancer
CTLA4: Cytotoxic T-Lymphocyte-Associated Protein 4
ATB: Antibiotics
HP-NAP: Helicobacter pylori neutrophil-activating protein
rMBP-NAP: Hp-NAP recombined with the maltose binding protein
Chi-rNap: HP-NAP-embedded Chitosan nanoparticles
OVs: Oncolytic viruses
CAR: Chimeric antigen receptor
